# Supplementary material for: Impact of Group Management and Transfer on Individual Sociality in Highland Cattle (Bos taurus)
Source: Front Vet Sci. 2019 Jun 12;6:183. doi: 10.3389/fvets.2019.00183 (PMC6581677; doi:10.3389/fvets.2019.00183)
Supplement: Supplementary file 1 [file Data_Sheet_1.docx]

Supplementary information for :

Impact of group management and transfer on individual sociality in Highland cattle (*Bos Taurus*)

Multi-model inferences and Node label permutations

For each GLMM, multi-model inferences were run to compare and rank candidate models according to (i) their respective Akaike Information Criterion (AIC) after correction for small sample sizes (AICc) and (ii) normalized Akaike weights (AICw) (Burnham and Anderson, 2004). Burnham and Anderson (2004) emphasized that information theoretic approaches (AIC) allow formal inference to be based on more than just one best model (lowest AIC) and lead to more robust conclusions. This means that for each combination of factors, all models were tested and ranked according to the best AIC. ΔAICc is the difference in AICc between a given model and the model with the lowest AIC. The AIC weight indicates the probability that a given model will be the best among candidate models. Models with a ΔAICc <10 were considered equally possible candidates, and their statistics were averaged. The null model (random effect: identity of individuals) was included as a possible candidate but was never among the models with lowest AICc. The results also indicate relative variable importance (RVI), which is the number of times a variable is present in the best models. Model inference and averaging were carried out with the R package ‘MuMIn’ (Bartoń, 2013). Node label permutations were also performed (Croft et al., 2011; Farine, n.d.). Permutations are a robust and modern standard way to compare statistical models based on the original observed data to a distribution of null models based on randomised data (Farine, 2013; Farine and Whitehead, 2015). After 1000 randomisations, the statistical parameters of interest (e.g. model estimates) of the models based on observed data were compared with “null” models based on randomised data. If a substantial proportion (95%) of the statistical parameters derived from models based on observed data were lower/higher than those derived from models based on randomised data, we could conclude that the observed effects on sociality were different from those expected to arise by chance. The randomisation procedure is exactly the same for all analyses. The P-values indicated in the tables are based on these permutation procedures.


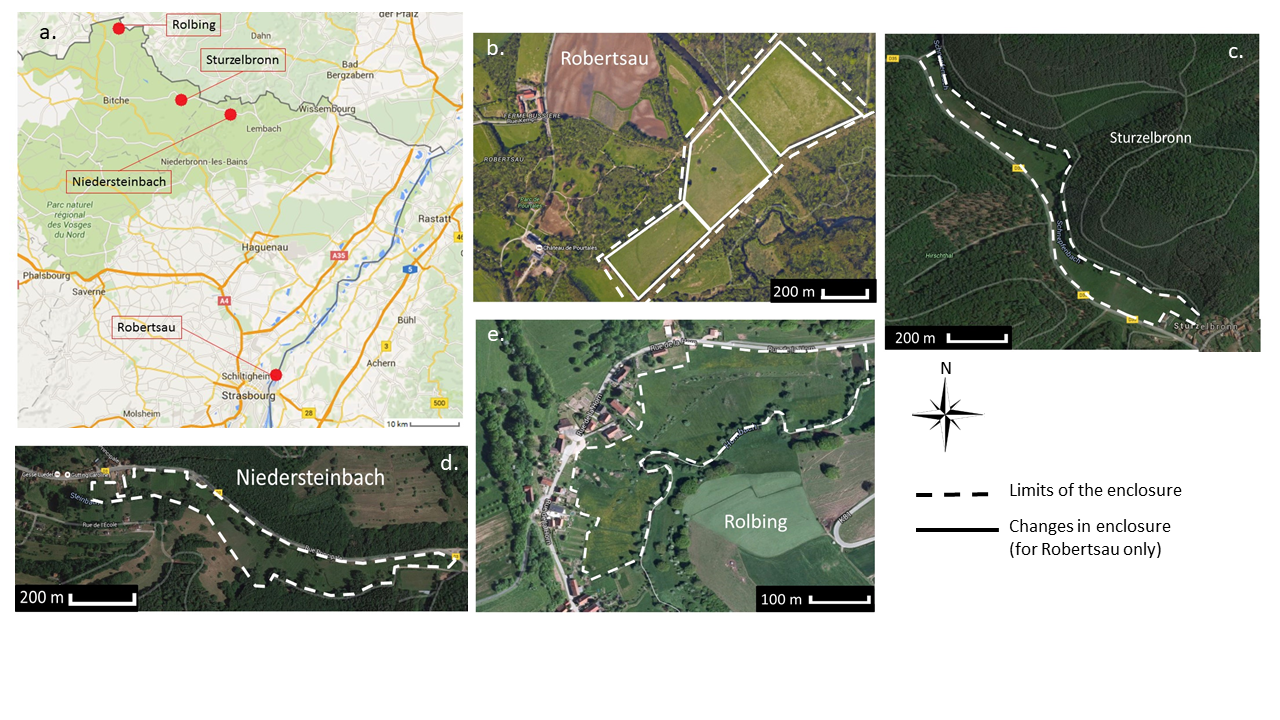


Figure S1: (a.) locations of the four different sites, (b.) Robertsau study site with the different enclosures (as full line), (c.) Sturzelbronn study site, (d.) Niedersteinbach study site and (e.) Rolbing study site.

Figure S2: Plots of residual normality distribution for the eigenvector centrality (a.), the strength of associations (b.), the difference of eigenvector centrality between two transfers (c.) and the difference of strength of associations between two transfers (d.).


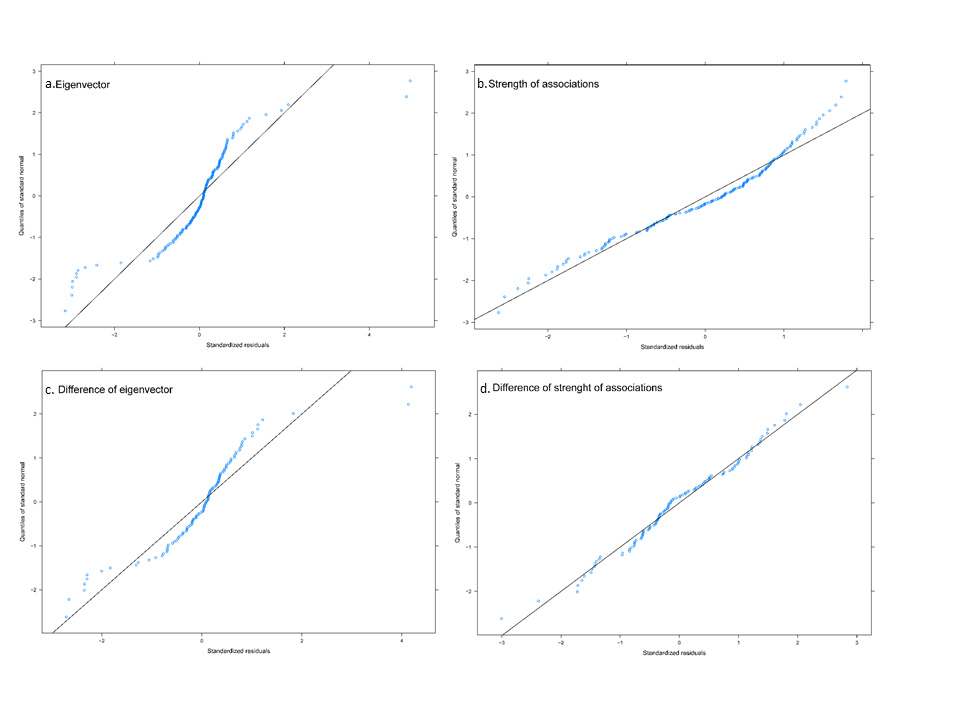


Table S1: Model selection table for the influence of socio-demographic factors on the eigenvector centrality. Models are ranked according to the best AIC. In bold, the models retained for the p-values average

| Model n° | (Int) | age | domin | famil | sex | df | logLik | AICc | ΔAIC | weight |
| --- | --- | --- | --- | --- | --- | --- | --- | --- | --- | --- |
| **1** | **0.381** |  |  |  |  | **3** | **194.6** | **-383** | **0** | **0.736** |
| **3** | **0.408** |  | **-0.05** |  |  | **4** | **194.4** | **-381** | **2.47** | **0.215** |
| **5** | **0.407** |  |  | **-0.03** |  | **4** | **192.5** | **-377** | **6.28** | **0.032** |
| **7** | **0.433** |  | **-0.05** | **-0.03** |  | **5** | **192.3** | **-374** | **8.9** | **0.009** |
| **2** | **0.368** | **0.004** |  |  |  | **4** | **190.8** | **-373** | **9.81** | **0.005** |
| 9 | 0.38 |  |  |  | + | 5 | 190.4 | -371 | 12.57 | 0.001 |
| 4 | 0.398 | 0.002 | -0.04 |  |  | 5 | 189.7 | -369 | 14 | 0.001 |
| 11 | 0.388 |  | -0.05 |  | + | 6 | 190.5 | -369 | 14.51 | 0.001 |
| 6 | 0.399 | 0.005 |  | -0.04 |  | 5 | 189 | -368 | 15.5 | 0 |
| 13 | 0.383 |  |  | -0.03 | + | 6 | 188.4 | -364 | 18.79 | 0 |
| 8 | 0.424 | 0.003 | -0.04 | -0.03 |  | 6 | 187.8 | -363 | 20.08 | 0 |
| 15 | 0.391 |  | -0.06 | -0.03 | + | 7 | 188.6 | -363 | 20.59 | 0 |
| 10 | 0.368 | 0.005 |  |  | + | 6 | 186.9 | -361 | 21.8 | 0 |
| 12 | 0.38 | 0.002 | -0.05 |  | + | 7 | 186 | -357 | 25.84 | 0 |
| 14 | 0.37 | 0.005 |  | -0.04 | + | 7 | 185.2 | -356 | 27.44 | 0 |
| 16 | 0.382 | 0.003 | -0.05 | -0.04 | + | 8 | 184.2 | -352 | 31.56 | 0 |

Table S2: Model selection table for the influence of socio-demographic factors on the strength of associations. Models are ranked according to the best AIC. In bold, the models retained for the p-values average. Effect of sex is indicated by + because it is a factor (categorical).

| Model n° | (Int) | age | domin | famil | sex | df | logLik | AIC | ΔAIC | weight |
| --- | --- | --- | --- | --- | --- | --- | --- | --- | --- | --- |
| **13** | **0.9447** |  |  | **0.2704** | **+** | **6** | **55.82** | **-99.6** | **0.0** | **0.707** |
| **14** | **0.9074** | **0.0147** |  | **0.2465** | **+** | **7** | **54.98** | **-96** | **3.7** | **0.113** |
| **5** | **0.6884** |  |  | **0.1959** |  | **4** | **51.77** | **-95.5** | **4.1** | **0.091** |
| **15** | **0.9531** |  | **-0.0583** | **0.2663** | **+** | **7** | **54.36** | **-94.7** | **4.9** | **0.06** |
| **7** | **0.7191** |  | **-0.0580** | **0.194** |  | **5** | **50.28** | **-90.6** | **9.1** | **0.008** |
| **1** | **0.8599** |  |  |  |  | **3** | **48.23** | **-90.5** | **9.2** | **0.007** |
| **16** | **0.9119** | **0.0139** | **-0.0172** | **0.246** | **+** | **8** | **52.92** | **-89.8** | **9.8** | **0.005** |
| 6 | 0.6651 | 0.0124 |  | 0.1749 |  | 5 | 49.70 | -89.4 | 10.2 | 0.004 |
| 9 | 0.9718 |  |  |  | + | 5 | 48.46 | -86.9 | 12.7 | 0.001 |
| 2 | 0.808 | 0.0155 |  |  |  | 4 | 47.38 | -86.8 | 12.9 | 0.001 |
| 10 | 0.9228 | 0.0181 |  |  | + | 6 | 49.18 | -86.4 | 13.3 | 0.001 |
| 3 | 0.8929 |  | -0.0658 |  |  | 4 | 46.91 | -85.8 | 13.8 | 0.001 |
| 8 | 0.6824 | 0.0113 | -0.0276 | 0.1755 |  | 6 | 47.76 | -83.5 | 16.1 | 0 |
| 11 | 0.9818 |  | -0.0736 |  | + | 6 | 47.42 | -82.8 | 16.8 | 0 |
| 4 | 0.8254 | 0.0143 | -0.0265 |  |  | 5 | 45.44 | -80.9 | 18.8 | 0 |
| 12 | 0.9282 | 0.0171 | -0.0211 |  | + | 7 | 47.19 | -80.4 | 19.3 | 0 |

Table S3: Model selection table for the influence of socio-demographic factors on the difference of eigenvector centralities after a transfer. Models are ranked according to the best AIC. In bold, the models retained for the p-values average

| Model n° | (Intrc) | age | domin | famil | nb.ind | df | logLik | AIC | ΔAIC | weight |
| --- | --- | --- | --- | --- | --- | --- | --- | --- | --- | --- |
| **1** | **-0.0096** |  |  |  |  | **3** | **102.32** | **-198.6** | **0.0** | **0.854** |
| **2** | **-0.0400** | **0.0092** |  |  |  | **4** | **100.56** | **-193.1** | **5.5** | **0.054** |
| **5** | **0.0073** |  |  | **-0.0185** |  | **4** | **100.40** | **-192.8** | **5.9** | **0.046** |
| **3** | **-0.0090** |  | **0.0171** |  |  | **4** | **100.17** | **-192.3** | **6.3** | **0.036** |
| 9 | -0.0107 |  |  |  | -0.0022 | 4 | 97.62 | -187.2 | 11.4 | 0.003 |
| 6 | -0.0308 | 0.0091 |  | -0.0099 |  | 5 | 98.57 | -187.1 | 11.5 | 0.003 |
| 7 | 0.0217 |  | 0.0278 | -0.0332 |  | 5 | 98.43 | -186.9 | 11.8 | 0.002 |
| 4 | -0.0418 | 0.0096 | -0.0146 |  |  | 5 | 98.41 | -186.8 | 11.8 | 0.002 |
| 13 | 0.0155 |  |  | -0.0287 | -0.0024 | 5 | 95.78 | -181.6 | 17.1 | 0 |
| 8 | -0.0396 | 0.0096 | -0.0138 | -0.0023 |  | 6 | 96.52 | -181 | 17.6 | 0 |
| 11 | -0.0102 |  | 0.0111 |  | -0.0021 | 5 | 95.43 | -180.9 | 17.8 | 0 |
| 10 | -0.0390 | 0.0088 |  |  | -0.0008 | 5 | 95.39 | -180.8 | 17.9 | 0 |
| 15 | 0.0275 |  | 0.0238 | -0.0409 | -0.0023 | 6 | 93.77 | -175.5 | 23.1 | 0 |
| 14 | -0.0255 | 0.0086 |  | -0.0143 | -0.0009 | 6 | 93.44 | -174.9 | 23.8 | 0 |
| 12 | -0.0408 | 0.0092 | -0.0155 |  | -0.0008 | 6 | 93.25 | -174.5 | 24.2 | 0 |
| 16 | -0.0340 | 0.0090 | -0.0130 | -0.0070 | -0.0009 | 7 | 91.39 | -168.8 | 29.9 | 0 |

Table S4: Model selection table for the influence of socio-demographic factors on the difference of strength of associations after a transfer. Models are ranked according to the best AIC. In bold, the models retained for the p-values average

| Model n° | Int. | Age | Domin | Famil | Nb.ind | df | logLik | AIC | ΔAIC | weight |
| --- | --- | --- | --- | --- | --- | --- | --- | --- | --- | --- |
| **7** | **-0.6639** |  | **0.242** | **0.7524** |  | **5** | **4.422** | **1.2** | **0.0** | **0.473** |
| **5** | **-0.7887** |  |  | **0.8802** |  | **4** | **3.352** | **1.3** | **0.1** | **0.441** |
| **6** | **-0.8732** | **0.0203** |  | **0.8992** |  | **5** | **1.985** | **6** | **4.9** | **0.041** |
| **13** | **-0.7529** |  |  | **0.8354** | **-0.0103** | **5** | **1.101** | **7.8** | **6.6** | **0.017** |
| **15** | **-0.6394** |  | **0.2252** | **0.7199** | **-0.0095** | **6** | **1.89** | **8.2** | **7.1** | **0.014** |
| **8** | **-0.7600** |  | **0.0150** | **0.1768** | **0.8009** | **6** | **1.884** | **8.2** | **7.1** | **0.014** |
| 14 | -0.8281 | 0.0158 |  | 0.8619 | -0.0076 | 6 | -1.279 | 14.6 | 13.4 | 0.001 |
| 16 | -0.7085 | 0.0102 | 0.1839 | 0.7581 | -0.0079 | 7 | -1.281 | 16.6 | 15.4 | 0 |
| 3 | 0.0318 |  | 0.4848 |  |  | 4 | -7.173 | 22.3 | 21.2 | 0 |
| 11 | 0.0244 |  | 0.4490 |  | -0.0125 | 5 | -8.839 | 27.7 | 26.5 | 0 |
| 4 | 0.0211 | 0.0031 | 0.4744 |  |  | 5 | -10.745 | 31.5 | 30.3 | 0 |
| 1 | 0.0156 |  |  |  |  | 3 | -14.542 | 35.1 | 33.9 | 0 |
| 12 | 0.0370 | -0.0038 | 0.4599 |  | -0.0130 | 6 | -12.375 | 36.8 | 35.6 | 0 |
| 9 | 0.0080 |  |  |  | -0.0155 | 4 | -15.18 | 38.4 | 37.2 | 0 |
| 2 | -0.0375 | 0.0160 |  |  |  | 4 | -17.066 | 42.1 | 41.0 | 0 |
| 10 | -0.0182 | 0.0081 |  |  | -0.0142 | 5 | -18.492 | 47 | 45.8 | 0 |

References:

Bartoń, K., 2013. MuMIn: multi-model inference. R Package Version 1.

Burnham, K.P., Anderson, D.R., 2004. Multimodel inference understanding AIC and BIC in model selection. Sociol. Methods Res. 33, 261–304.

Croft, D.P., Madden, J.R., Franks, D.W., James, R., 2011. Hypothesis testing in animal social networks. Trends Ecol. Evol. 26, 502–507. https://doi.org/10.1016/j.tree.2011.05.012

Farine, D.R., 2013. Animal social network inference and permutations for ecologists in R using asnipe. Methods Ecol. Evol. 4, 1187–1194. https://doi.org/10.1111/2041-210X.12121

Farine, D.R., n.d. A guide to null models for animal social network analysis. Methods Ecol. Evol. n/a-n/a. https://doi.org/10.1111/2041-210X.12772

Farine, D.R., Whitehead, H., 2015. Constructing, conducting and interpreting animal social network analysis. J. Anim. Ecol. 84, 1144–1163.
